# Whole-genome sequence and resistance determinants of four *Elizabethkingia anophelis* clinical isolates collected in Hanoi, Vietnam

**DOI:** 10.1038/s41598-024-57564-3

**Published:** 2024-03-27

**Authors:** Florian Commans, Juliette Hayer, Bich Ngoc Do, Thi Thanh Tam Tran, Thi Thu Hang Le, Thanh Thuyet Bui, Huu Song Le, Anne-Laure Bañuls, Tien Sy Bui, Quang Huy Nguyen

**Affiliations:** 1 LMI DRISA, University of Science and Technology of Hanoi (USTH), Vietnam Academy of Science and Technology (VAST), Hanoi, Vietnam; 2 UMR MIVEGEC (University of Montpellier- IRD-CNRS), Montpellier, France; 3 Department of Microbiology, 108 Military Central Hospital, Hanoi, Vietnam; 4 Vietnamese-German Center for Medical Research, 108 Military Central Hospital, Hanoi, Vietnam

**Keywords:** Biotechnology, Evolution, Genetics, Microbiology

## Abstract

Four isolates of the opportunistic pathogen *Elizabethkingia anophelis* were identified for the first time in a Vietnamese hospital and underwent antimicrobial susceptibility testing and genomic characterization by whole-genome sequencing. Complete, fully circularized genome sequences were obtained for all four isolates. Average Nucleotide Identity analysis and single nucleotide polymorphism phylogenetic analysis on the core genome showed that three of the four isolates were genetically distinct, ruling out the hypothesis of a single strain emergence. Antibiotic susceptibility testing highlighted multi-resistant phenotypes against most antimicrobial families, including beta-lactams, carbapenems, aminoglycosides, quinolones, macrolides, amphenicols, rifamycins and glycopeptides. Additionally, in silico genomic analysis was used to correlate the phenotypic susceptibility to putative resistance determinants, including resistance genes, point mutations and multidrug efflux pumps. Nine different resistance genes were located inside a single resistance pocket predicted to be a putative Integrative and Conjugative Element (ICE). This novel ICE was shared by three isolates from two different lineages and displayed similarity with ICEs previously reported in various *Elizabethkingia* and *Chryseobacterium* species. The role of such ICEs in pathogenicity, genome plasticity and antimicrobial resistance gene spread within the *Flavobacteriaceae* family needs to be further elucidated.

## Introduction

The emerging pathogen *Elizabethkingia anophelis,* a Gram-negative, aerobic, non-motile rod-shaped bacterium, is a clinically-relevant member of the *Elizabethkingia* genus from the *Flavobacteriaceae* family ^1–3^. To date, this genus comprises eight distinct species. Among them, *Elizabethkingia meningoseptica*, *Elizabethkingia miricola* and *E. anophelis* have been the most studied due to their clinical relevance, while the other species have rarely been associated with nosocomial infections ^4^. All members of the *Elizabethkingia* genus are mainly considered to be environmental bacteria because they are ubiquitous in various natural reservoirs such as soil, freshwater bodies and insect or amphibian guts ^5–7^. *E. anophelis* is commonly found in the midgut of different mosquito species, including the malaria vectors *Anopheles gambiae* and *Anopheles stephensi* ^8–13^, and has already been detected from animal food products such as raw bovine milk ^14^. Infections caused by *E. anophelis* tend to be rare but problematic, usually displaying lethality rates of 23.5–33.3% ^15–17^ and up to 70% ^18^. The high lethality rates are partially due to its intrinsic resistance to several antimicrobial families, notably most β‐lactams (including carbapenems) and aminoglycosides, as well as its frequent resistance to other families (e.g. tetracyclines, chloramphenicol and fluoroquinolones) ^2,15,19–21^. Clinical manifestation of *E. anophelis* includes meningitis, pneumoniae, bloodstream and urinary tract infections, endophthalmitis or sepsis ^3,15–18,22,23^. Neonates, elderly people and people with underlying co-morbidities, such as cancer, diabetes mellitus, compromised immunity, chronic medical conditions or COVID-19 infection, are at higher risk of infection ^15,17,24–28^. *E. anophelis* transmission and global epidemiology remain unclear. Nevertheless, its frequent detection in various areas of the hospital environment (e.g. sinks, faucets, mechanical ventilation equipment, medical devices, healthcare worker hands) and its ability to colonize and persist over long periods of time in water systems (such as tap water pipes) highlight the potential for sporadic outbreak emergence in hospitals ^7,19,29^. In the last decade, *E. anophelis* has been confirmed as the responsible agent of several outbreaks across the world, including in Singapore ^25^, the United States ^23,30^, Hong Kong ^15^ and Taiwan ^31,32^. Here, we report for the first time the detection of four multi-resistant *E. anophelis* clinical isolates in a hospital of Hanoi, Vietnam. We carried out antimicrobial susceptibility testing and whole-genome sequencing (short and long reads) to obtain four complete, fully circularized genome sequences.

## Material and methods

### Bacterial isolates and clinical data collection

In 2020–2021, a cluster of suspected *Elizabethkingia* infections was detected at the Clinical Institute of Infectious Diseases and the Intensive Care Center and Poison Control of the 108 Military Central Hospital in Hanoi Capital, which is one of the five largest general hospitals in Vietnam (2200 beds and 5000 outpatients/day). Sampling and bacterial isolation were part of the routine laboratory procedures at the Microbiology Department. All procedures were performed in accordance with the ethical standards of 108 Military Central Hospital and the 1964 Helsinki declaration and its later amendments. Therefore, formal consent was not required for this study. Four Vietnamese patients were hospitalized for different reasons: one had been admitted following a traumatic traffic accident (NVH72, aged 55 years old), while the three others (NVB490, NNN508 and VTKC53, aged 67, 84 and 76 years old, respectively) suffered from pre-existing comorbidities that led to multiple secondary infections (including fungal infection, bilateral pneumonia, sepsis and UTI). Four clinical isolates (EAV_NVB490, EAV_NNN508, EAV_VTKC53, and EAV_NVH72) were cultured from bronchial fluid (n = 1) and from sputum (n = 3) on Blood Agar culture medium (Oxoid, UK) at 37 °C. After isolation, isolates were initially identified as *E. meningoseptica* using the VITEK-2 Compact system (BioMérieux, Inc., NC, USA) when antibiotic susceptibility testing was performed, and as *E. anophelis* using the VITEK-MS system (BioMérieux, Inc., NC, USA). They were then stored at − 80 °C in Tryptic Soy Broth medium with 50% glycerol, and were subsequently sent to the University of Science and Technology of Hanoi (USTH) for additional genomic characterization. Upon arrival, the four isolates were confirmed to be *E. anophelis* by 16S rRNA sequencing using the primer pairs 8F (5′-AGAGTTTGATCCTGGCTCAG-3′) and 518R (5′-ATTACCGCGGCTGCTGG-3′), followed by whole-genome sequencing (see “Whole-genome sequencing, assembly and annotation”).

### Antibiotic susceptibility testing

First, the Minimum Inhibitory Concentrations (MIC) of ticarcillin, ticarcillin/clavulanic acid, piperacillin/tazobactam, cefotaxime, cefepime, ceftazidime, imipenem, meropenem, aztreonam, gentamicin, tobramycin, amikacin, kanamycin, ciprofloxacin, norfloxacin, levofloxacin, trimethoprim/sulfamethoxazole and colistin were obtained at the 108 Military Central Hospital for therapeutic purposes using the VITEK-2 Compact system (AST Card, BioMérieux, Inc., NC, USA) following the manufacturer’s instructions. The MIC of colistin was also determined using microdilution method. Antibiotic susceptibilities were interpreted according to the standard guidelines for ‘Other Non-Enterobacteriaceae’ from the Clinical and Laboratory Standards Institute (CLSI M100 30th edition, 2020) ^33^. Upon arrival at USTH, the Kirby-Bauer Disk Diffusion Susceptibility Test (DDT) was used with 43 antibiotics (Thermo Scientific, UK): ampicillin (10 μg), penicillin G (1UI), oxacillin (5 μg), temocillin (30 μg), ticarcillin (75 μg), amoxicillin/clavulanic acid (20/10 μg), ticarcillin/clavulanic acid (75/10 μg), piperacillin/tazobactam (100/10 μg), cefotaxime (30 μg), cefoxitin (30 μg), cefepime (30 μg), ceftazidime (30 μg), cefpodoxime (10 μg), cephalexin (30 μg), imipenem (10 μg), meropenem (10 μg), ertapenem (10 μg), aztreonam (30 μg), gentamicin (10 μg), tobramycin (10 μg), amikacin (30 μg), kanamycin (30 μg), netilmicin (10 μg), ciprofloxacin (5 μg), norfloxacin (10 μg), levofloxacin (5 μg), ofloxacin (5 μg), nalidixic acid (30 μg), trimethoprim/sulfamethoxazole (1.25/23.75 μg), colistin (10 μg), tetracycline (30 μg), minocycline (30 μg), erythromycin (15 μg), chloramphenicol (30 μg), rifampicin (5 μg), vancomycin (5 μg), teicoplanin (30 μg), fusidic acid (10 μg), fosfomycin (200 μg), linezolid (10 μg), pristinamycin (15 μg), clindamycin (2 μg) and lincomycin (15 μg) following the method described in the CLSI guidelines (CLSI M02, 13th edition, 2018). The CLSI inhibition zone diameter interpretive criteria were not available for ‘*Other Non-Enterobacteriaceae’*. Therefore, the CLSI standards from *Acinetobacter* spp. species were used, except for penicillin, norfloxacin, erythromycin, chloramphenicol, rifampicin, vancomycin, teicoplanin, fosfomycin and linezolid, for which the standards of *Enterococcus* spp. were adapted according to Chiu et al*.* ^34^. No susceptibility interpretation was attempted for antibiotics for which neither *Acinetobacter* spp. nor *Enterococcus* spp. displayed informative inhibition zone diameters (including lincosamides, streptogramins, fusidanes, cefoxitin and the amoxicillin-clavulanic acid combination).

### Whole-genome sequencing, assembly and annotation

Genomic DNA from the four clinical strains was extracted using the DNeasy PowerLyzer Microbial Kit (Qiagen, Hilden, Germany) following the manufacturer’s instructions. For short-read sequencing, DNA samples were sent to Beijing Genomics Institute (BGI Group, Shenzhen, China), where libraries were constructed and whole-genome sequencing was performed on a BGI-SEQ500 platform using a 2 × 150 paired-end protocol. For long-read sequencing, the Rapid Barcoding Kit (SQK-RBK004) from Oxford Nanopore Technology was used to generate libraries that were loaded and sequenced on a R9.4.1 flow cell (FLO-MIN106D) with the MinION M1kB device (ONT, Oxford, United Kingdom). Base calling, demultiplexing, quality check and trimming were performed by BGI (short reads) and with the default MinKNOW and guppy software programs (long reads). Hybrid de novo genome assembly, combining short and long reads, was carried out with Unicycler v0.4.9 ^35,36^ using default parameters. Statistics about the resulting assemblies were obtained using QUAST v5.1.0 ^37^, and assembly completeness (based on the presence of housekeeping genes for the *Flavobacteriales* lineage) was checked with BUSCO v5.2.2 ^38^. Genomic contamination scores were estimated using CheckM v.1.2.2 ^39^. The four genomes were then annotated with Prokka v1.14.6 ^40^ and the prokaryotic genome Rapid Annotations using Subsystems Technology (RAST) Server ^41–43^. Clustered Regularly Interspaced Short Palindromic Repeat (CRISPR)/CRISPR-associated protein (Cas) systems were detected using CRISPRCasFinder v4.2.2 ^44^. Putative antimicrobial resistance genes were predicted in silico using a combination of tools including ResFinder v4.2.3 ^45^, RGI and the Comprehensive Antibiotic Resistance Database (CARD) v3.1.4 ^46^, and manual inspection of the .gbk files from Prokka and RAST annotations. Point mutations were detected by manually aligning the relevant gene sequences against the corresponding sequences of the reference *E. anophelis* R26 (NZ_CP023401.1).

### Comparative genome analysis

ANI values were estimated with OrthoANI v.1.2, a stand-alone program based on the OrthoANI algorithm using orthologous fragment pairs to calculate nucleotide identities ^47^. The genome sequences of the four isolates were compared pairwise with the genomes of a few representative species within the *Elizabethkingia* genus, including *E. meningoseptica* CSID_3000516977 (NZ_MAHI00000000.1)*, E. miricola* EM_CHUV(NZ_LIQC00000000.1), *E. ursingii* CSID_3000516135 (NZ_MAHB00000000.1), *E. occulta* G4070 (NZ_MAHX00000000.1), *E. argenteiflava* YB22 (NZ_JAAABJ000000000.1) and *E. bruuniana* FDAARGOS_1031 (NZ_CP067018.1), and with five complete *E. anophelis* reference genomes isolated in various locations worldwide, namely CSID_3015183684 (NZ_CP015066.2), FMS-007 (NZ_CP006576.1), JM-87 (NZ_MAGY00000000.1), NUHP1 (NZ_CP007547.1) and R26 (NZ_CP023401.1). These genomes were chosen because they were complete and represented a range of relevant environmental or clinical isolates from various places of the world (including USA, Singapore, China and Sweden). We added as well the sequence of *Chryseobacterium* spp. POL2 (NZ_CP049298.1), another member of the *Flavobacteriaceae* family and a sister-group to *Elizabethkingia* spp. All genome sequences were downloaded from the National Center for Biotechnology Information (NCBI) genome repository. A cut-off value of 95% identity was used for species delimitation (i.e. the ANI threshold traditionally used for microbial taxonomy) ^48^. The ANI matrix resulting from the OrthoANIu tool was visualized with CIMMiner (https://discover.nci.nih.gov/cimminer/) and the “equal width” binning method to generate a color-coded heatmap of genomic similarities between genome pairs ^49^.

The Harvest suite ^50^ was used for core-genome alignment and visualization of a phylogenetic tree based on a Single Nucleotide Polymorphism (SNP)-calling within the core genome. The suite includes the Parsnp tool, highly efficient to align the core-genome of closely related species, and the dynamic visual platform Gingr, used to explore the trees and alignments generated by Parsnp ^50^. Three Vietnamese isolates were included in the analysis (EAV_NVB490, EAV_VTKV53 and EAV_NVH72) along with 29 sequences of other *E. anophelis* isolates, i.e. the totality of the complete genomes that were available on the NCBI repository at the time of writing. The strain CSID_3000521207 (NZ_CP015067.2) was used as the reference core genome. The complete list of the isolates included in the analysis is available in Supplementary Table S1.

### Detection and visualization of mobile genetic elements

The genomic islands of the four Vietnamese isolates were predicted by IslandViewer 4 ^51^, using the .gbk files annotated by RAST as input. IslandViewer uses a combination of different methods (IslandPick, IslandPath-DIMOB, SIGI-HMM) to detect genomic regions thought to have horizontal origins, including sequence composition (codon bias, GC content), sequence prediction (mobility genes) and comparative genomics (regions not present in related strains). The prediction of putative Integrative and Conjugative Elements (ICE) was confirmed by manual inspection of the annotation files from prokka and RAST, looking for genes involved in conjugative machineries (such as *tra* genes, transposases, relaxases, integrases or Type 4 Coupling Proteins) or direct repeats (DR) flanking the integrative element. Linear portions of some ICE were visualized through the Linear Genome Plot tool from Galaxy Version 1.0 by uploading sequencing data on the Galaxy web platform and using the public server (https://cpt.tamu.edu/galaxy-pub) to analyze them ^52,53^.

## Results

### Antimicrobial susceptibility testing

The VITEK-2 Compact system (AST card) or/and the Diffusion Susceptibility Test (DDT) method were used to test the susceptibility of the four Vietnamese isolates (EAV_NVB490, EAV_NNN508, EAV_VTKC53 and EAV_NVH72) to 43 antibiotics or antibiotic combinations. The results are detailed in Table 1. Overall, the four *E. anophelis* isolates were multidrug resistant, as they displayed a resistant phenotype to at least one drug in three or more antimicrobial families. With the VITEK2 Compact system, isolates were resistant to nearly all tested antibiotics: all beta-lactams (including penicillins, cephalosporins, carbapenems and monobactams), fluoroquinolones, aminoglycosides and polymyxins. The only exception was isolate EAV_NVB490 that showed intermediate resistance to the trimethoprim-sulfamethoxazole combination. Similarly, with the DDT method, the four isolates were resistant to nearly all beta-lactams (except the piperacillin-tazobactam combination), fluoroquinolones, aminoglycosides (but not isolate EAV_VTKC53 to amikacin), nalidixic acid, colistin, chloramphenicol and fosfomycin. Conversely, they were sensitive to minocycline and the piperacillin-tazobactam and trimethoprim-sulfamethoxazole combinations. All isolates showed intermediate resistance to teicoplanin, and two isolates (EAV_NVB490 and EAV_NNN508) to vancomycin and tetracycline. Unlike the other three isolates, EAV_VTKC53 was sensitive to amikacin and tetracycline, and was only moderately resistant to erythromycin and rifampicin. Moreover, its growth was inhibited in the presence of ticarcillin, cefoxitin, cefepime, imipenem, gentamicin, levofloxacin and chloramphenicol (asterisks in Table 1). This growth defect did not result in an inhibition zone diameter wide enough to be considered as sensitive, but was larger compared with the other isolates.

**Table 1:** Antimicrobial susceptibility testing of EAV_NVB490, EAV_NNN508, EAV_VTKC53 and EAV_NVH72 using the VITEK2 Compact system (MIC, in mg/L; left) and DDT (inhibition zone diameter, in mm; right).

	VITEK2 MIC (mg/L)				DDT inhibition diameter (mm)			
	EAV_NVB490	EAV_NNN508	EAV_VTKC53	EAV_NVH72	EAV_NVB490	EAV_NNN508	EAV_VTKC53	EAV_NVH72
Penicillins								
Ampicillin					0 (R)	0 (R)	0 (R)	0 (R)
Penicillin G					0 (R)	0 (R)	0 (R)	0 (R)
Oxacillin					0	0	0	0
Temocillin					0	0	0	0
Ticarcillin	> 128 (R)	> 128 (R)			0 (R)	0 (R)	09 (R)*	0 (R)
Beta-lactam/Beta-lactamase inhibitor combinations								
Amoxicillin/clavulanic acid					14	13	15	18
Ticarcillin/clavulanic acid	> 128 (R)	> 128 (R)			0 (R)	0 (R)	07 (R)*	0 (R)
Piperacillin/tazobactam	> 128 (R)	> 128 (R)	> 128 (R)	> 128 (R)	**23 (S)**	**21 (S)**	**23 (S)**	**22 (S)**
Cephalosporins								
Cefotaxime			> 64 (R)	> 64 (R)	0 (R)	0 (R)	0 (R)	0 (R)
Cefoxitin					0	0	13*	0
Cefepime	> 64 (R)	> 64 (R)	> 64 (R)	> 64 (R)	0 (R)	0 (R)	14 (R)*	0 (R)
Ceftazidime	> 64 (R)	> 64 (R)	> 64 (R)	> 64 (R)	0 (R)	0 (R)	0 (R)	0 (R)
Cefpodoxime					0	0	0	0
Cephalexin					0	0	0	0
Carbapenems								
Imipenem	> 16 (R)	> 16 (R)	> 16 (R)	> 16 (R)	0 (R)	0 (R)	09 (R)*	0 (R)
Meropenem	> 16 (R)	> 16 (R)	> 16 (R)	> 16 (R)	0 (R)	0 (R)	0 (R)	0 (R)
Ertapenem					0	0	0	0
Monobactams								
Aztreonam	> 64 (R)	> 64 (R)			0	0	0	0
Aminoglycosides								
Gentamicin	> 16 (R)	> 16 (R)	> 16 (R)	> 16 (R)	0 (R)	0 (R)	10 (R)*	0 (R)
Tobramycin	> 16 (R)	> 16 (R)			0 (R)	0 (R)	0 (R)	0 (R)
Amikacin	> 64 (R)	> 64 (R)	> 64 (R)	> 64 (R)	0 (R)	0 (R)	20 (S)*	0 (R)
Kanamycin	> 256 (R)	> 256 (R)	> 256 (R)	> 256 (R)	0	0	0	0
Netilmicin					0	0	0	0
Fluoroquinolones								
Ciprofloxacin	> 4 (R)	> 4 (R)	> 4 (R)	> 4 (R)	0 (R)	0 (R)	0 (R)	0 (R)
Norfloxacin			> 16 (R)	> 16 (R)	0 (R)	0 (R)	0 (R)	0 (R)
Levofloxacin	> 8 (R)	> 8 (R)			0 (R)	0 (R)	08 (R)*	0 (R)
Ofloxacin					0	0	0	0
Nalidixic acid					0	0	0	0
Folate pathway inhibitors								
Trimethoprim-sulfamethoxazole	**40 (I)**	80 (R)	> 320 (R)	> 160 (R)	**18 (S)**	**17 (S)**	**16 (S)**	**22 (S)**
Polymyxins								
Colistin**	> 16 (R)	> 16 (R)	> 16 (R)	> 16 (R)	0	0	0	0
Tetracyclines								
Tetracycline					**13 (I)**	**13 (I)**	18 (S)*	10 (R)
Minocycline					**25 (S)**	**25 (S)**	31 (S)*	**23 (S)**
Macrolides								
Erythromycin					0 (R)	0 (R)	22 (I)*	0 (R)
Amphenicols								
Chloramphenicol					0 (R)	0 (R)	1 (R)*	0 (R)
Rifamycins								
Rifampicin					09 (R)	09 (R)	19 (I)*	09 (R)
Glycopeptides								
Vancomycin					**16 (I)**	**16 (I)**	12 (R)	13 (R)
Teicoplanin					**13 (I)**	**11 (I)**	**13 (I)**	**12 (I)**
Fusidanes								
Fusidic Acid					12	14	20*	15
Phosphonic acids								
Fosfomycin					0 (R)	0 (R)	0 (R)	0 (R)
Oxazolidinones								
Linezolid					18 (R)	18 (R)	19 (R)	**21 (I)**
Streptogramins								
Pristinamycin					16	17	22	20
Lincosamides								
Clindamycin					22	20	26	30
Lincomycin					0	0	0	14

When possible, the interpretation is indicated between brackets; no interpretation was attempted in the absence of CLSI interpretive guidelines for *Acinetobacter* spp. or *Enterococcus* spp. Intermediate (I) and sensitive (S) phenotypes are indicated in bold, but not resistant (R) phenotypes. In DDT, some diameters were marked with an asterisk (*) when the antimicrobial agent had a visible inhibitory effect on bacterial growth, but the inhibition zone was not large enough to consider it as sensitive/intermediate resistant.

**MIC of colistin: microdilution assay.

Comparison of the results obtained with the two testing methods highlighted several discrepancies. For example, the trimethoprim-sulfamethoxazole and piperacillin-tazobactam combinations inhibited bacterial growth in the DDT but not in the VITEK2 Compact assay. Similarly, the EAV_VTKC53 isolate was sensitive to amikacin in the DDT assay, but not in the VITEK2 Compact assay. Overall, the resistance profile variability was higher with the DDT assay than with the VITEK2 Compact assay, in which virtually all tested antibiotics displayed high MIC values except for the SXT with EAV_NVB490.

### Genome sequences

The complete, circular genome sequences of the four Vietnamese isolates EAV_NVB490, EAV_NNN508, EAV_VTKC53 and EAV_NVH72 were obtained by hybrid de novo assembly. The genome lengths, completeness, GC content and number of coding sequences detected with the two annotation tools (Prokka and RAST) are summarized in Table 2. Genome sizes varied between ~ 4.12 and 4.17 Mb, and completeness scores were > 97% using the *Flavobacteriales* lineage as reference. Two clustered regularly interspaced short palindromic repeat (CRISPR)/CRISPR-associated protein (CRISPR/Cas) systems were identified in the EAV_VTKC53 isolate by CRISPRCasFinder, and none in the other three isolates. The four complete, circularized genome sequences and the raw reads have been deposited in the *European Nucleotide Archive (ENA)* at EMBL-EBI under the bioproject accession number PRJEB49667 (https://www.ebi.ac.uk/ena/browser/view/PRJEB49667). The isolates EAV_NVB490, EAV_NNN508, EAV_VTKC53 and EAV_NVH72 are represented by the biosamples SAMEA12009938, SAMEA112835271, SAMEA12009939, SAMEA12009940 and the nucleotide accession numbers GCA_927611585.2, GCA_951394135.2, GCA_927611525.2 and GCA_927611535.2, respectively.

**Table 2:** Genome assemblies of EAV_NVB490, EAV_NNN508, EAV_VTKC53, and EAV_NVH72.

	EAV_NVB490	EAV_NNN508	EAV_VTKC53	EAV_NVH72
Genome length	4,139,197	4,139,199	4,120,123	4,167,098
Status	Complete	Complete	Complete	Complete
GC content (%)	35.8	35.8	35.64	35.79
Completeness (%)	97.1	97.1	97.2	97
CDS [prokka]	3820	3818	3767	3849
CDS [RAST]	3910	3909	3858	3952
Total RNA	63	63	62	62
tRNA	53	53	52	52
tmRNA	1	1	1	1
CRISPR/Cas systems	0	0	2	0
Genome contamination	2.38%	2.38%	5.72%	3.03%

### Comparative genomics analysis

**Figure 1:**
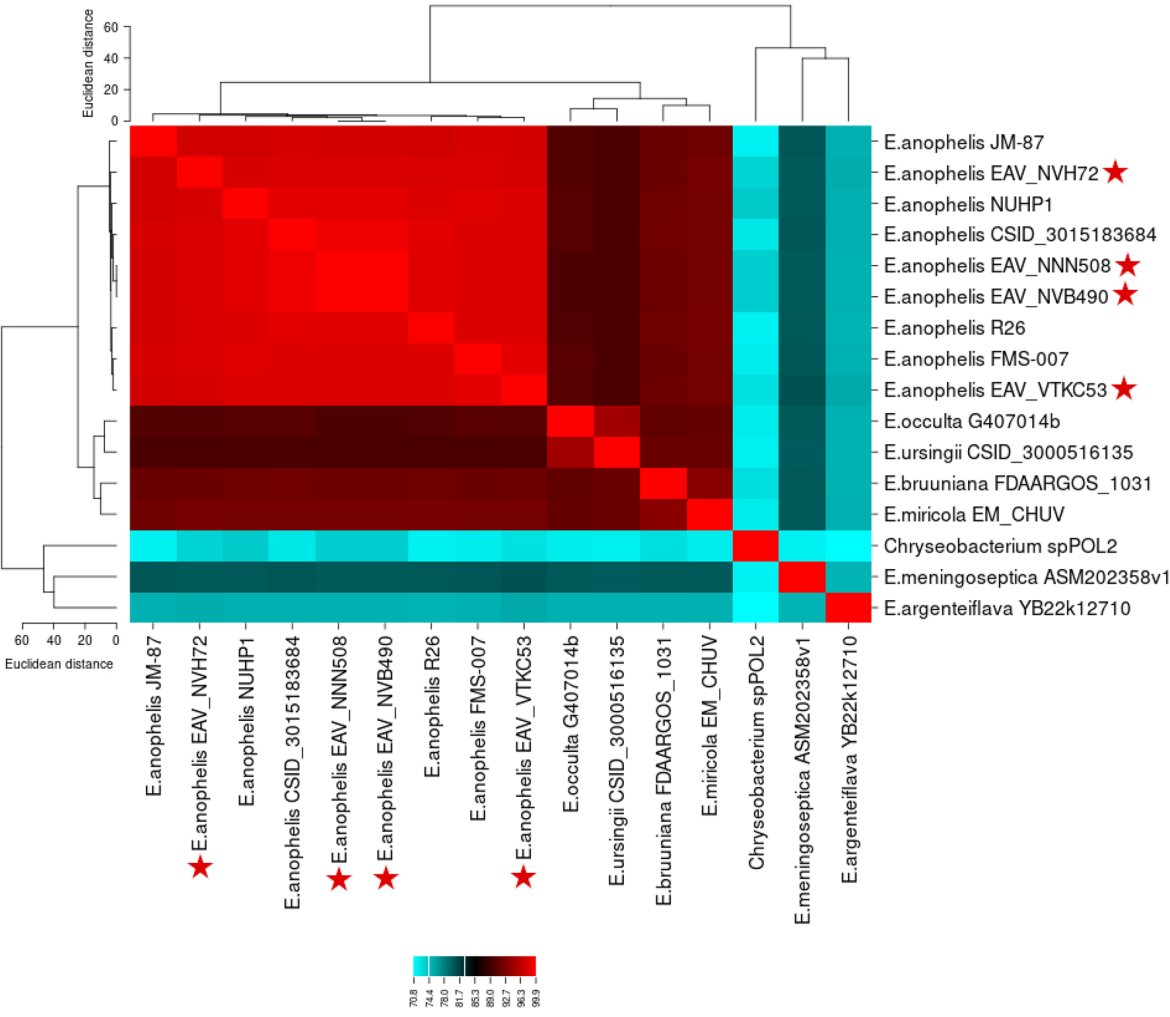
Heatmap showing the ANI values obtained for each pairwise genome comparison. ANI values ranged from 70.80% (light blue) to 99.99% (bright red). A cluster tree displaying the Euclidian distance between sequences is shown on top and on the right. Red stars indicate the four Elizabethkingia anophelis isolates sequenced in this study.

To confirm the species-level identification of the four isolates and assess their genetic relatedness, we calculated their genome sequence identity (ANI values) relative to the reference genomes of five *E. anophelis* strains from various origins in the world, and seven closely related species amongst the *Elizabethkingia* genus and the *Flavobacteriaceae* family. The heatmap constructed with the ANI values obtained for each intra- and inter-species pairwise comparison is in Fig. 1 and the matrix of raw values obtained with OrthoANI for each genome pair is in Supplementary Table S2. Using a 95% cut-off value, all four Vietnamese isolates (tagged with a red star in Fig. 1) were confirmed to be *E. anophelis* (values > 97% with any of the other five *E. anophelis* strains used as reference). Overall, the ANI values ranged from 70.8 to 99.9% and classified the genomes into three clusters. *E. anophelis* was clearly separated from all other species, with ANI values between 89 and 92% (for *E. occulta*, *E. miricola*, *E. bruuniana* and *E. ursingii* that are more closely related) and between 70.8 and 89% (for *Chryseobacterium* sp.POL2, *E. meningoseptica* and *E. argenteiflava* that are more distant). The isolates EAV_NVB490 and EAV_NNN508 displayed an ANI value of 99.99% between them, indicating that they were representing a single strain ^54^. However, they displayed ANI values of 97.90% and 98.10% when compared with EAV_NVH72 and EAV_VTKC53.

**Figure 2:**
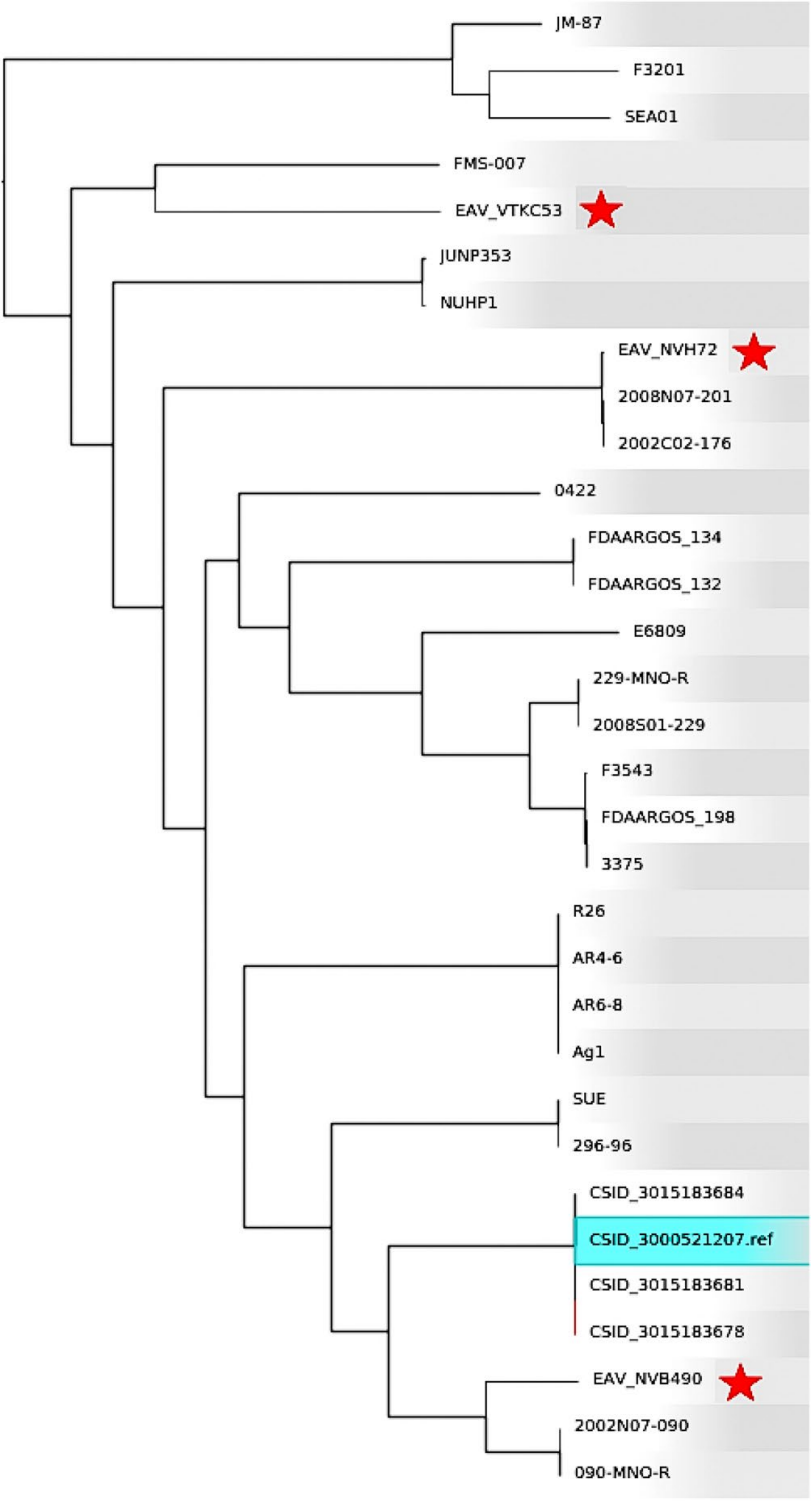
Core-genome Single Nucleotide Polymorphisms (cgSNP)-based phylogenetic tree of the Vietnamese isolates EAV_NVB490, EAV_VTKC53 and EAV_NVH72, along with 29 other E. anophelis complete sequences. The strain CSID_3000521207, used as the reference genome, is highlighted in light-blue in the tree, and the three Vietnamese isolates are tagged with a red star.

The relationships of the Vietnamese isolates with 29 other *E. anophelis* genomes were further investigated through core-genome SNP-calling based phylogeny (Fig. 2). Isolate EAV_NNN508 was excluded from the analysis, as it represented the same strain as EAV_NVB490. In the generated phylogenetic tree, EAV_NVB490 was closely related to isolates 2002N07-090 and 090-MNO-R (both recently isolated in Taiwan) and to the cluster of strains CSID (implicated in the Wisconsin outbreak of 2015–2016). EAV_VTKC53 was closer to FMS-007 (isolated in China in 2013) and EAV_NVH72 of 2008N07-201 and 2002C02-176, two other Taiwanese isolates. Overall, Fig. 2 strongly suggests that EAV_NVB490/NNN508, EAV_VTKC53 and EAV_NVH72 originate from separate lineages and have distinct phylogenetic origins.

### In silico prediction of resistance determinants and correlation with AST

The genome sequences were used to predict the genetic determinants of antimicrobial resistance in the four *E. anophelis* isolates by integrating the results of the different annotation and prediction tools. Table 3 lists the identified genes, point mutations and multidrug efflux pumps that could be implicated in the multidrug resistance phenotype of the four isolates. This analysis identified 49, 49, 38, and 52 resistance determinants in EAV_NVB490, EAV_NNN508, EAV_VTKC53 and EAV_NVH72, respectively. Among all the antibiotic resistance genes identified in the four isolates, seven families of genes were involved in resistance to beta-lactams, four in resistance to aminoglycosides, two in resistance to macrolides, tetracyclines and amphenicols, and one in resistance to rifamycins and glycopeptides. Moreover, we identified multiple mutations that could inhibit the action of quinolones and folate pathway inhibitors, as well as a total of 17 different families of efflux pump genes potentially involved in antibiotic transport across the membrane of the four isolates.

**Table 3:** Prediction of antibiotic resistance determinants in EAV_NVB490, EAV_NNN508, EAV_VTKC53 and EAV_NVH72, including efflux pumps, point mutations and chromosomally-encoded resistance genes.

	EAV_NVB490	EAV_NNN508	EAV_VTKC53	EAV_NVH72	Annotation	Predicted resistance phenotype
β-lactams						
blaB	+(blaB-1)	+(blaB-1)	+(blaB-11)	+(blaB-29)	Subclass B1 metallo-beta-lactamase, type II	Amoxicillin, ampicillin, ticarcillin, piperacillin, combinations with beta-lactamase inhibitors, Imipenem, Meropenem
blaGOB	+(blaGOB-20)	+(blaGOB-20)	+(blaGOB-6)	+(blaGOB-38)	Subclass B3 metallo-beta-lactamase	Amoxicillin, ampicillin, cefoxitin, ceftazidime, ertapenem, imipenem, meropenem
blaCME	+	+	+	+	Class A extended-spectrum serine-beta-lactamase	Ampicillin, piperacillin, cefazolin, ceftazidime, ceftriaxone, aztreonam
blaOXA-10	+	+	–	+	Class D beta-lactamase OXA-10-like	Unknown carbapenem
ccrA	+	+	–	+	Subclass B1 metallo-beta-lactamase, type II	Unknown beta-lactam
blaPER-1_a	–	–	–	+	Subclass A2 extended-spectrum beta-lactamase PER-1	Unknown beta-lactam
blaPER-1_b	+	+	+	+	Class A beta-lactamase PER-1	Unknown beta-lactam
Aminoglycosides						
aadK_a	–	–	–	+	AadS family aminoglycoside-N(6′)-adenylyltransferase	Streptomycin
aadK_b	+	+	+	+	AadS family aminoglycoside-N(6′)-adenylyltransferase	Streptomycin
aacC1	+	+	–	+	GNAT family gentamicin-N(3′)-acetyltransferase	Gentamicin
aacA4	+	+	–	+	GNAT family aminoglycoside-N(6')-acetyltransferase	Amikacin, kanamycin, gentamicin
Quinolones						
gyrA mutation(s)	+(Ser83Ile)*	+(Ser83Ile)*	+(Ser83Ile)*(Glu282Asp)(Ala841Val)(Ile842Ala)	+(Ser83Ile)*(Asp829His)	DNA gyrase subunit A; quinolone resistance-determining region (QRDR)	Levofloxacin, ciprofloxacin, ofloxacin
Folate pathway inhibitors						
folP mutation(s)	+(Leu10Ile)(Thr52Ala)*(Asn171Asp)*(Leu174Ile)	+(Leu10Ile)(Thr52Ala)*(Asn171Asp)*(Leu174Ile)	+(Leu10Ile)(Thr52Ala)*(Phe180Leu)	+(Leu10Ile)(Thr52Ser)*(Met88Ile)(Ile94Val)(Thr147Lys)(Met163Ile)*(Asn171Asp)*(Thr214Ala)(Tyr261His)	Dihydropteroate synthase; sulfonamide resistance-determining region	Sulfamethoxazole
Tetracyclines						
tet(X)_a	+	+	–	+	Flavin-dependent monooxygenase, tetracycline resistance protein TetX	Tetracycline
tet(X)_b	+	+	+	+	Flavin-dependent monooxygenase, tetracycline resistance protein TetX	Tetracycline
Macrolides						
mef(C)	+	+	–	+	Macrolide resistance protein, MFS efflux pump Mef(C)	Erythromycin
mph(G)	+	+	–	+	Macrolide 2'-phosphotransferase, Mph(E)/Mph(G) family	Erythromycin
Amphenicols						
cat_a	+	+	–	+	Type B chloramphenicol-O-acetyltransferase CatB	Chloramphenicol
cat_b	+	+	+	+	Type B chloramphenicol-O-acetyltransferase CatB	Chloramphenicol
Rifamycins						
arr	+	+	–	+	Rifampin ADP-ribosyl transferase	Rifampicin
Glycopeptides						
vanW	+	+	+	+	Vancomycin resistance protein W	Vancomycin
Multidrug efflux transporters						
emrA	+^3^	+^3^	+^3^	+^3^	HlyD family secretion protein, tripartite multidrug resistance system	Multi-drugs, colistin
macA/B	+^2^	+^2^	+^2^	+^2^	ABC transporter, macrolide export protein	Multidrug, erythromycin
acrA	+	+	+	+	RND family efflux transporter	Multi-drugs
bepE = adeF	+^7^	+^7^	+^7^	+^6^	RND family efflux transporter	Multi-drugs
emrE	+	+	+	+^2^	SMR family efflux transporter	Multi-drugs
emrD	+	+	+	+	MFS family efflux transporter	Multi-drugs
norM	+	+	+	+	MATE family efflux transporter	Multi-drugs
stp	+	+	+	+^2^	MFS family efflux transporter	Multi-drugs
mexA	+^2^	+^2^	+^3^	+^3^	RND family efflux transporter	Multi-drugs
mdtC	+^3^	+^3^	+^2^	+^2^	CusA/CzcA, RND family efflux transporter	Multi-drugs, acriflavine
yheI	+	+	+	+	ABC family transporter, ATP-binding protein	Multi-drugs
ybhR	+	+	+	+	ABC family transporter permease	Multi-drugs
lnrL/lptB/ybhF	+	+	–	+	ABC family transporter ATP-binding protein	Multi-drugs, linearmycin
yedA	+^2^	+^2^	+^2^	+^2^	EamA family transporter	Unknown
yicL	+	+	+	+	EamA family transporter	Unknown
marC	+	+	+	+	MarC family protein	Multi-drugs
mepA	+	+	–	+	MATE family efflux transporter	Multi-drugs

A short description of the products and the expected resistance phenotypes are also indicated. When relevant, the exact genomic location of a point mutation or the allelic version of a gene (for the blaB and blaGOB families) is given between brackets. The point mutations marked with an asterisk (*) have already been reported to be involved in resistance ^16,59,60^. For multidrug efflux pumps, the number of genes identified with this name and annotation in the genome is given between brackets, although they might represent non-identical sequences and therefore different proteins of the same family.

All four isolates harbored at least two chromosome-encoded metallo-β-lactamases (MBL), one from the *bla*_*B*_ family (subclass B1) and one from the *bla*_*GOB*_ family (subclass B3), as well as two class A extended-spectrum β-lactamases (ESBL) (*bla*_*CME*_ and *bla*_*PER1*_). Intrinsic resistance to beta-lactams within the *Elizabethkingia* genus is well documented and has been related to the presence of these MBL: BlaB and GOB provide resistance to carbapenems, while CME could be involved in resistance to cephalosporins and monobactams ^55,56^. *Elizabethkingia* spp. members are also the only known bacteria that carry several MBL genes encoded in their chromosome ^21^. These MBL display some degree of diversity with an underestimated number of allelic versions in the *Elizabethkingia* genus ^21^. A BLAST analysis of the *bla* sequences of the four Vietnamese isolates highlighted the presence of the *bla*_*B-1*_, *bla*_*B-11*_ and *bla*_*B-29*_, and *bla*_*GOB-20*_, *bla*_*GOB-6*_ and *bla*_*GOB-38*_ subtypes in EAV_NVB490/NNN508, EAV_VTKC53, and EAV_NVH72, respectively. Additionally, EAV_NVB490, EAV_NNN508 and EAV_NVH72 carried the type II MBL *ccrA* (subclass B1) and the β-lactamase *bla*_*OXA-10*_ (class D), and EAV_NVH72 harbored a unique subclass A2 ESBL. Concerning genes related to resistance to aminoglycosides, *aad*K was detected in all four isolates, while *aac*C1 and *aac*A4 were in EAV_NVB490, EAV_NNN508 and EAV_NVH72, and an additional version of the gene *aad*K was detected in EAV_NVH72 only. Since EAV_VTKC53 was the only isolate sensitive to amikacin in DDT, it reinforces the hypothetic involvement of *aac*A4 in amikacin resistance. Two *tet(*X*)* genes (encoding two different flavin-dependent monooxygenases that inactivates enzymatically tetracyclines) ^57^ were identified. The *tet(*X*)* gene has already been detected sporadically in various species of the *Flavobacteriaceae* family, the potential ancestral source of this tetracycline destructase ^58^. EAV_VTKC53, which unlike the others harbored a single version (and not two) of the *tet*(X) gene, was the only isolate sensitive to tetracycline, with a significantly larger inhibition zone than the other three isolates in DDT. Additionally, the three other isolates also carried the macrolide-resistance tandem genes *mef*C and *mph*G that might be involved in the observed resistance to erythromycin: *mph*(G) is a phosphotransferase that can inactivate macrolides by phosphorylation, and *mef*(C) is an efflux pump involved in high-level macrolide resistance ^59^. It has been shown that the introduction of these tandem genes in *E. coli* significantly increased the MIC values for several macrolides ^59^. EAV_VTKC53, which does not carry these two genes, had a significantly larger inhibition zone diameter compared with the other three isolates (23 mm vs. 0 mm), although this could only be interpreted as an intermediately resistant phenotype. The rifampicin-resistance gene *arr*, detected in EAV_NVB490, EAV_NNN508 and EAV_NVH72, is a class I ADP-ribosyltransferase that inactivates rifampicin ^60^. Arr enzymes are widespread in bacteria and have already been identified in the *Bacteroidota* phylum ^61^. EAV_NVB490, EAV_NNN508 and EAV_NVH72 were resistant to rifampicin but EAV_VTKC53, which lacks *arr*, was only intermediately resistant. Moreover, these three isolates harbored two *cat* genes previously detected in the *E. anophelis* NUHP1, and encoding a type B chloramphenicol-acetyltransferase that is strongly suspected to provide resistance to chloramphenicol by catalyzing its acetylation ^62^. Last, all four isolates harbored a vancomycin-resistance gene containing a *van*W*-*like domain that could provide partial or complete resistance to glycopeptides ^63^. However, vancomycin usually does not display significant activity against Gram-negative bacteria due to their lipidic bilayer cell membrane.

Concerning single-point mutations, all four isolates carried the Ser83Ile mutation in *gyr*A (encoding the gyrase subunit A) that confers potential resistance to fluoroquinolones ^17,64^, and which could explain the observed resistance to ciprofloxacin, norfloxacin, levofloxacin and ofloxacin. For sulfonamides, several point mutations (including Leu10Ile, Thr52Ala, Thr52Ser, Asn171Asp and Met163Ile) were detected in the *fol*P gene that encodes a dihydropteroate synthase, an enzyme necessary for the folate reduction pathway and in which an accumulation of mutations is suspected to increase resistance to folate pathway inhibitors such as the trimethoprim-sulfamethoxazole combination ^65^. In this study, resistance to SXT was observed in the VITEK2 Compact assay but not in DDT. Among the four isolates, EAV_NVH72 displayed the highest number of *fol*P mutations (n = 9), including three mutations involved in sulfonamide resistance (asterisks in Table 3).

Lastly, approximately 30 putative efflux pump-encoding genes were detected in each isolate, predicted to be multiple copies of 17 different multidrug resistance transporters (Table 3). Several of the main families of membrane transporters were represented, including the major facilitator superfamily (MFS), the ATP-binding cassette (ABC) superfamily, the multidrug and toxic-compound extrusion (MATE) family, the small multidrug resistance (SMR) family and the resistance-nodulation-division (RND) family. Although it has not been demonstrated that all these pumps can transport antibiotics, they could contribute at least partly to the observed multi-resistant phenotype of the *E. anophelis* isolates.

### Detection of putative mobile genetic elements

**Figure 3:**
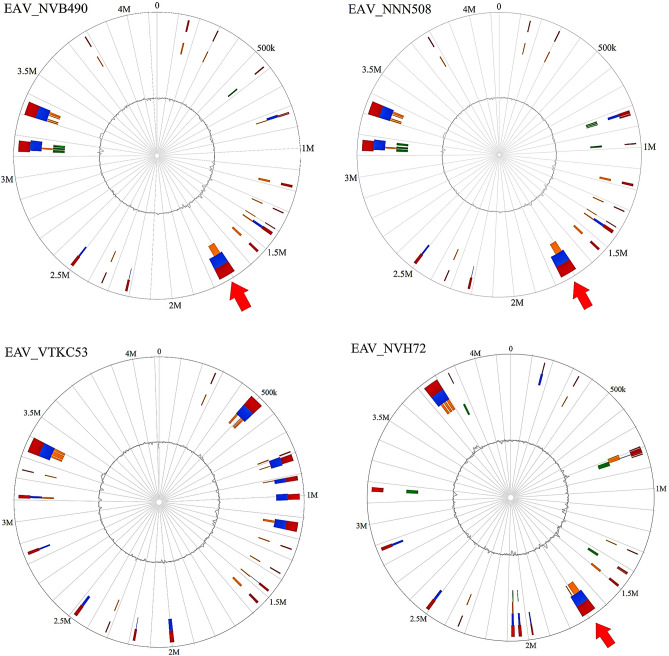
Circular visualization of the Genomic Islands (GI) predicted for the four Vietnamese isolates EAV_NVB490, EAV_NNN508, EAV_VTKC53 and EAV_NVH72. The blocks are colored according to the prediction method: IslandPick (green), IslandPath-DIMOB (blue), SIGI-HMM (orange), and the integrated results are in dark red. The light red arrow highlights the putative Integrative & Conjugative Elements ICEEaIII(NVB490/NNN508) and ICEEaIII(NVH72).

**Figure 4:**
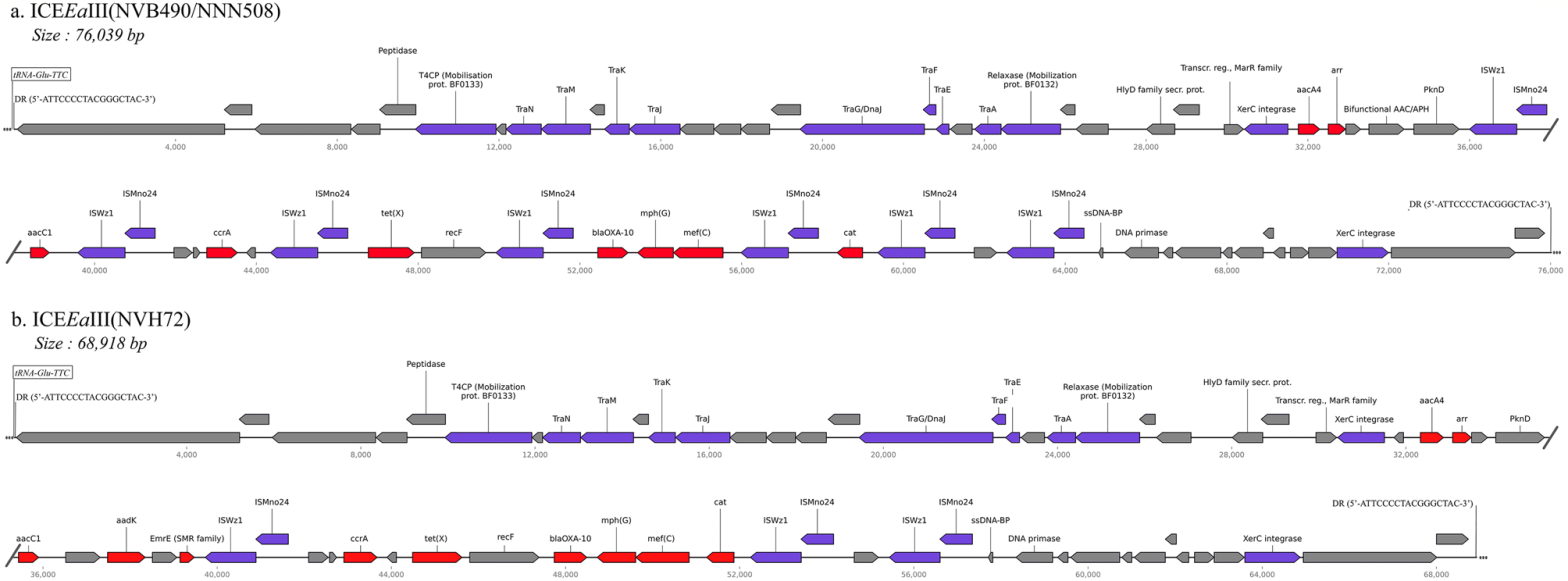
Linear map of the genetic structure of the putative Integrative and Conjugative Elements (**a**) ICEEaIII(NVB490/NNN508) and (**b**) ICEEaIII(NVH72). The genes involved in antimicrobial resistance are tagged in red; the genes involved in the ICE conjugative machinery or in genetic mobility are tagged in purple; the other genes (including predicted proteins without annotation) are tagged in grey.

Following assembly and annotation, we predicted genomic islands (GI) and other putative mobile genetic elements (MGE) in the four Vietnamese isolates using IslandViewer (Fig. 3). Three GI larger than 50,000 bp were detected by at least two different prediction methods in isolate EAV_NVB490/NNN508, and two others were predicted in EAV_VTKC53 and in EAV_NVH72. EAV_VTKC53 also harbored four GI of intermediate size (between 20,000 and 50,000 bp). One of the large predicted GI (tagged by a red arrow in Fig. 3) seemed to be located at a very similar location in the genome sequences of both EAV_NVB490/NNN508 and EAV_NVH72, but was absent in EAV_VTKC53. Moreover, nine different antibiotic resistance genes previously described (*ccr*A, *bla*_*OXA-10*_, *aac*C1, *aac*A4, an additional *aad*K for EAV_NVH72, *tet*(X), *cat*, *mef*C*, mph*G and *arr*) were harbored in this region, ranging from 1,715,338 to 1,742,564 bp in EAV_NVB490/NNN508 and from 1,688,044 to 1,707,580 bp in EAV_NVH72. This close arrangement of multiple resistance genes was indicative of the presence of an Integrative and Conjugative Element (ICE), a type of MGE integrated in the chromosome whose circulation in *Elizabethkingia* spp. has already been discussed ^66,67^. This hypothesis was supported by the identification of a wide range of genes encoding a putative conjugative machinery in the immediate proximity, including an integrase, a relaxase, a Type 4 Coupling Protein and a Type 4 Secretion System formed by multiple Tra proteins (see the annotation files from bioproject PRJEB49667, under accession numbers OX596081.1, OX596082.1, OX596083.1 and OX596084.1). These genes can be used as markers for the detection of putative ICE, because this machinery is required for the ICE excision out of the chromosome and its re-integration inside a new chromosome after transfer via conjugation to a novel recipient strain. It is also their structural arrangement, the type of excision and conjugation mechanism and the integration target site that defines the type of the ICE, as described by Xu et al.^66^ in their classification of the ICE of *E. anophelis* into the three types I, II and III. A linear map of this newly discovered putative ICE in EAV_NVB490/NNN508 and in EAV_NVH72 is illustrated in Fig. 4. The structure and the cargo genes were quite similar in the three isolates, despite some minor differences (e.g. the presence of the additional *aadK* gene in EAV_NVH72). Furthermore, the architecture of the modular genes was very similar to the type III described in ^66^, featuring seven *tra* genes (traAEGJKMN) flanked by the relaxase (annotated as Mobilization protein BF0132) and the T4CP (annotated as Mobilization protein BF0133). However, an additional gene *tra*F was present between *tra*E and *tra*G in both versions of the ICE detected here. They also shared the same integration site, a gene coding for a transfer RNA (tRNA), in this case specifically a tRNA-Glu-TTC gene. Their integrase was a tyrosine recombinase annotated as XerC, present in two copies. Also, a tandem of transposase genes annotated as ISWz1 and ISMno24 of the IS91 family was present in multiple copies in both ICE (seven times in ICE*Ea*III(NVB490/NNN508) and three times in ICE*Ea*III(NVH72)).The presence of this conjugative machinery suggests that this ICE is still capable to excise itself to spread to other bacteria through conjugation. Until further characterisation, the two ICEs were named ICE*Ea*III(NVB490/NNN508) and ICE*Ea*III(NVH72), based on their predicted type and the code of the associated isolate. Their size was estimated at 76,039 bp and 68,918 bp, respectively, thanks to the identification of a common direct repeat sequence of 17 bp (5′-ATTCCCCTACGGGCTAC-3′) that marked the beginning and the end of the ICE in all three genomes.

## Discussion

In this study, we presented the antibiotic susceptibility profile and genomic characterization of four clinical *E. anophelis* isolates identified at 108 Military Central Hospital, Hanoi. To our knowledge, this is the first report of *E. anophelis* isolated in Vietnam and the first time that complete, fully circularized genomic sequences are obtained in the country. However, several outbreaks of *E. anophelis* have been reported over the last decade in the region ^68,69^, including in Taiwan ^26^, Hong Kong ^15^, Shanghai ^70^ and Fujian ^71^. Moreover, *Elizabethkingia* spp. have already been detected in several South-East Asian countries (Singapore, Malaysia, Indonesia, China, Thailand and Cambodia), but never in the Philippines, Brunei, Myanmar, Laos and Timor-Leste ^68^. In Vietnam, Hoa and Hai recently reported the detection of an *E. meningoseptica* isolate in a patient who underwent liver transplantation at the 108 Military Central Hospital ^72^, but this is the only previous report of an *Elizabethkingia* spp. infection in Vietnam, and genome sequencing was not carried out to confirm species identity.

Here, the four isolates were initially identified as *E. meningoseptica* using the VITEK-2 Compact, and were later re-assigned to *E. anophelis*. For many years, the default databases of several mass spectroscopy identification systems, including VITEK-MS and Bruker MALDI Biotyper (Bruker Daltonics, Germany), could not accurately distinguish members of the *Elizabethkingia* genus, leading to the false-positive detection of *E. meningoseptica* in most cases ^15,73^. Recent global retrospective sequencing analyses of archived clinical specimens showed that *Elizabethkingia* spp*-*linked bacteremia was predominantly caused by *E. anophelis* and not by *E. meningoseptica* ^17,73–75^. From our study, we reiterate the need to update the default databases of current automated identification systems for *Elizabethkingia* spp. as well as the crucial role of sequencing-based methods for reliable clinical diagnostics.

The four Vietnamese patients suffered upon admission from multiple secondary infections (including pneumonia, sepsis and UTI). The routes of transmission of this pathogen remain poorly understood, but infections are mostly detected in patients with underlying conditions and a prolonged hospital stay ^76^. Even though no obvious source of *E. anophelis* contamination has been identified in this study, we suspect the infection by opportunistic *E. anophelis* to have occurred during their hospital stay, due to the pre-existing co-morbidities and a weakened immune system. Therefore, we cannot exclude the persistence of at least three different *E. anophelis* populations in the Hanoian hospital environment, leading to a risk of occasional nosocomial transmission.

The isolates EAV_NVB490 and EAV_NNN508 presented similar phenotypic resistance profiles, almost identical genome sizes (4,139,197 bp and 4,139,199 bp), and identical GC content, *bla*_B_ and *bla*_GOB_ alleles, point mutations and predicted resistant determinants. Moreover, these two isolates were from samples collected from two patients in adjacent units of the Clinical Institute of Infectious Diseases only five days apart (02/04/2021 and 07/04/2021). Therefore, we hypothesized that these two isolates might represent a single strain, probably resulting from an intra-hospital contamination. The ANI analysis indicated that all four isolates belonged to *E. anophelis* and that EAV_NVB490 and EAV_NNN508 shared a sequence similarity of 99.99%, confirming that these two genome sequences represented a single strain shared by two patients ^54^. Conversely, EAV_NVB490/EAV8_NNN508 shared only 97.90% similarity with EAV_NVH72 and 98.10% similarity with EAV_VTKC53, suggesting distinct origins. The core-genome SNP analysis (cgSNP) confirmed that these three isolates belonged to separate lineages of the generated phylogenetic tree, ruling out the hypothesis of a single strain emergence. It should be noted that the cgSNP phylogenetic analysis includes only essential genes that are mostly inherited vertically; hence, their mutations rate is lower and confers a stronger signal-to-noise ratio for inferring phylogeny ^50^. This type of analysis is therefore more reliable than ANI and quite suitable to the *Elizabethkingia* genus, because the *Flavobacteriaceae* species are subject to frequent genomic re-arrangements due to a large diversity of ICE providing a high degree of genome plasticity.

Overall, the four Vietnamese *E. anophelis* isolates were resistant to most of the tested antibiotic families. A multi-resistant phenotype is not uncommon within the *Elizabethkingia* spp., and many studies already reported *E. anophelis* intrinsic resistance to different antibiotic families, such as beta-lactams and aminoglycosides ^3,15,17,22,70^. Conversely and in agreement with our findings, *E. anophelis* is susceptible to minocycline, trimethoprim-sulfamethoxazole and piperacillin-tazobactam. Therefore, these antibiotics are the first choice for multi-resistant *E. anophelis* infections because most strains exhibit sensitivity to at least one of these combinations ^17,23,26,70,73,75^. Importantly, we observed multiple discrepancies between the VITEK2 Compact and the DDT results. The poor concordance between testing methods in the *Elizabethkingia* genus has already been discussed ^34,77^. Susceptibilities determined by disk diffusion, gradient diffusion (E-test) and other automated methods often do not agree with the MIC values obtained using the broth dilution method, especially for some antibiotics including trimethoprim-sulfamethoxazole, piperacillin, vancomycin, tigecycline and ciprofloxacin ^34^. As dilution-based methods (e.g. broth microdilution) are considered the gold standard for antimicrobial susceptibility testing in *E. anophelis*, the DDT results should be considered less reliable and should be interpreted with caution, especially for therapeutic purposes ^77^.

The interpretation of the antimicrobial susceptibility testing data was limited also by the fact that neither established breakpoints (MIC values) nor inhibition zone diameter guidelines (disk diffusion assays) are available for *Elizabethkingia* spp. This lack of standard interpretation criteria has not been addressed yet. Traditionally, antibiotic susceptibility of *Elizabethkingia* spp. has been reported based on the MIC breakpoints of ‘*Other Non-Enterobacteriaceae*’ and on the inhibition zone diameters of *Acinetobacter* spp. and *Enterococcus* spp. from the Clinical and Laboratory Standards Institute and/or European Committee on Antimicrobial Susceptibility Testing guidelines. However, this is far from optimal and could be misleading. Caution is required when defining *Elizabethkingia* spp. strains as resistant, intermediate resistant or sensitive because this interpretation is not based on validated criteria and might not reflect the biological reality. We used the traditional standard guidelines for indicative purposes, but these interpretations might be less relevant than the overall differences in inhibition zone diameters among isolates.

Following sequencing and assembly, we used genomic data to correlate the AST observations with putative resistance determinants. Even though the lack of interpretive guidelines and the unreliable results of disk diffusion antibiotic sensitivity testing in *Elizabethkingia* spp. are serious limitations, our results clearly showed that EAV_VTKC53 had a much more sensitive phenotype compared with the other three isolates. Indeed, EAV_VTKC53 was sensitive to amikacin and tetracycline, and displayed intermediate resistance to erythromycin and rifampicin. Moreover, a visible growth inhibition effect and/or a significantly larger inhibition zone diameter was observed for eight additional antibiotics (ticarcillin, cefoxitin, cefepime, imipenem, gentamicin, levofloxacin, chloramphenicol and fusidic acid). Although the inhibition zone diameters were not large enough to characterize EAV_VTKC53 as sensitive to these agents, this isolate was the only one that did not harbor the additional genes potentially involved in resistance to cephalosporins (*ccr*A), carbapenems (*bla*_*OXA-10*_), aminoglycosides (*aac*C1, *aac*A4, additional *aad*K for EAV_NVH72), tetracyclines (additional *tet*(X)), amphenicols (additional *cat*), macrolides (*mef*C*, mph*G) and rifamycin (*arr*).

The genomic analysis of these specific genes showed that they were all localized in a small part of the chromosome that matched a predicted GI in EAV_NVB490/NNN508 and EAV_NVH72 sequences. Therefore, we hypothesized the presence of an ICE shared by all isolates except EAV_VTKC53, and providing additional resistance to several antimicrobial agents. The discovery of a complete conjugative machinery (including seven *tra* genes, a T4CP, a relaxase and an integrase) as well as a 17 bp direct repeat (DR) sequence flanking both ends of the ICE further reinforced this hypothesis. Interestingly, this same DR was previously identified in the ICE*Csp*POL2 in *Chryseobacterium* sp POL2 (NZ_CP049298.1), another species of the *Flavobacteriaceae* family closely related to *Elizabethkingia* spp. ^78^. Moreover, several genes from the cargo of ICE*Csp*POL2 were also identified inside the putative ICE*Ea*III(NVB490/NNN508) and ICE*Ea*III(NVH72), including *tet*(X), *cat*, the tandem *mph*(G)/*mef*(C) and *bla*_*OXA-10*_ genes. The two new ICEs also displayed structural similarity with several other ICEs type III previously discovered in the *Flavobacteriaceae* family, including ICE*Ea*III(5) in *E. anophelis* NUHP1 (NZ_CP007547.1) and ICE*Ea*III(10) in *E. anophelis* NUH6 (ASYJ00000000.1), in addition to ICE*Csp*POL2 ^78^. Many antibiotic resistance genes described in this study such as *aad*K, *ccr*A, cat or *tet*(X) were reported as well in these ICE, indicating a possible common origin or horizontal gene transfers having taken place between these strains. Interestingly, Fu et al. demonstrated that the ICE*Csp*POL2 in *Chrysebobacterium* could be transferred experimentally to an *Elizabethkingia* recipient, highlighting the potential involvement of ICE in the inter-species circulation of antimicrobial resistance genes ^78^. Because very few plasmids (as opposed to numerous ICEs) have been identified in the *Elizabethkingia* and the *Chryseobacterium* genera, we hypothesize that the involvement of chromosome-integrated genetic elements in the circulation of AMR might have been underestimated, and that ICEs might act as the main vehicle for the transfer of resistance genes (and possibly as well for genes involved in virulence or pathogenicity) within the *Flavobacteriaceae* family. An example from this study is the detected version of the carbapenem-resistance gene *bla*_*OXA-10*_, which was previously reported in ICE*Csp*POL2 but had never been reported in *E. anophelis* before. The newly discovered ICE*Ea*III(NVB490/NNN508) and ICE*Ea*III(NVH72), as well as other ICEs present in the four Vietnamese isolates, need to be better characterized to precisely describe their architecture, their transmission mechanism and their transfer frequency, and to trace back their evolutionary history and their circulation worldwide.

## Conclusion

This study reported for the first time the detection and isolation of four pathogenic *E. anophelis* in Vietnam. All four isolates displayed high resistance levels against most of the main antimicrobial agent families; however, the discrepancies observed between antibiotic susceptibility testing methods emphasized the need of appropriate interpretative guidelines for *Elizabethkingia* spp., especially for clinical decision-making. As this opportunistic pathogen usually displays high lethality rates, its increasingly multi-resistant phenotype is a worrying feature that needs to be closely monitored. A wide range of resistance determinants were detected by WGS analysis, including point mutations, resistance genes and multidrug efflux pumps. However, they were not investigated experimentally and their involvement in *E. anophelis* resistance phenotype should be precisely assessed. The detection of nine different resistance genes as cargo genes in a single ICE structure suggests that these mobile elements might represent an underestimated vehicle for spreading antimicrobial resistance genes within the *Elizabethkingia* genus. Two similar versions of this ICE were shared by three isolates of different phylogenetic origins and included resistance genes that had never been reported before in *Elizabethkingia* spp. Consequently, we cannot exclude the possibility of ICE transmission events that could have occurred between closely related members of the *Flavobacteriaceae* family. The description of this mobile element has therefore serious implications in terms of global public health, because ICE might play a key role in the future emergence of outbreaks of multi-resistant *E. anophelis* strains that would be difficult to address. Worldwide monitoring and the use of WGS approaches are critically needed to better understand the spread of the different *E. anophelis* lineages and the circulation of their mobile genetic elements.

### Supplementary Information

Supplementary Table S1.

Supplementary Table S2.

## Data Availability

All data analyzed in this study are included in this published article. The four complete *E. anophelis* genome sequences obtained and the raw reads were deposited in the European Nucleotide Archive (ENA) at EMBL-EBI under bioproject accession number PRJEB49667 (https://www.ebi.ac.uk/ena/browser/view/PRJEB49667).
